# A Study on Wheel Member Condition Recognition Using 1D–CNN

**DOI:** 10.3390/s23239501

**Published:** 2023-11-29

**Authors:** Jin-Han Lee, Jun-Hee Lee, Chang-Jae Lee, Seung-Lok Lee, Jin-Pyung Kim, Jae-Hoon Jeong

**Affiliations:** 1 Busan Transportation Corporation, Busan 47353, Republic of Korea; jin2023145@humetro.busan.kr (J.-H.L.); puafc@humetro.busan.kr (C.-J.L.); lesr42@humetro.busan.kr (S.-L.L.); 2 School of Software Engineering, Kunsan National University, Gunsan 54150, Republic of Korea; zmdzmd0541@naver.com; 3 Global Bridge Co., Ltd., Incheon 21990, Republic of Korea

**Keywords:** recognizing condition algorithm, machine learning, deep learning, wheel, tire

## Abstract

The condition of a railway vehicle’s wheels is an essential factor for safe operation. However, the current inspection of railway vehicle wheels is limited to periodic major and minor maintenance, where physical anomalies such as vibrations and noise are visually checked by maintenance personnel and addressed after detection. As a result, there is a need for predictive technology concerning wheel conditions to prevent railway vehicle damage and potential accidents due to wheel defects. Insufficient predictive technology for railway vehicle’s wheel conditions forms the background for this study. In this research, a real-time tire wear classification system for light-rail rubber tires was proposed to reduce operational costs, enhance safety, and prevent service delays. To perform real-time condition classification of rubber tires, operational data from railway vehicles, including temperature, pressure, and acceleration, were collected. These data were processed and analyzed to generate training data. A 1D–CNN model was employed to classify tire conditions, and it demonstrated exceptionally high performance with a 99.4% accuracy rate.

## 1. Introduction

The Busan Metro Line 4 is a light-rail system in South Korea that connects Minam Station in Oncheon–dong, Dongnae–gu, Busan, to Anpyeong Station in Anpyeong–ri, Chulma–myeon, Gijang County, Busan. It is the first commercial light-rail system in South Korea and was implemented as a public project. One notable feature of this light-rail system is that it uses rubber tires for its wheels, distinguishing it from conventional heavy rail systems that typically use steel wheels. Rubber tires offer advantages such as precise positioning, rapid acceleration and deceleration, and the ability to navigate tight curves. As a result, it is expected that rubber-tired wheels will be installed on most new light-rail routes in South Korea, instead of traditional steel wheels used in heavy rail systems.

Rubber tires are highly susceptible to wear and tear, with frequent issues such as uneven wear and cracking, making them less durable compared to steel wheels. Furthermore, the inspection of tires heavily relies on the subjective judgment of maintenance personnel, leading to a lack of consistency in the criteria for diagnosing tire conditions and determining when to replace them.

**Figure 1:**
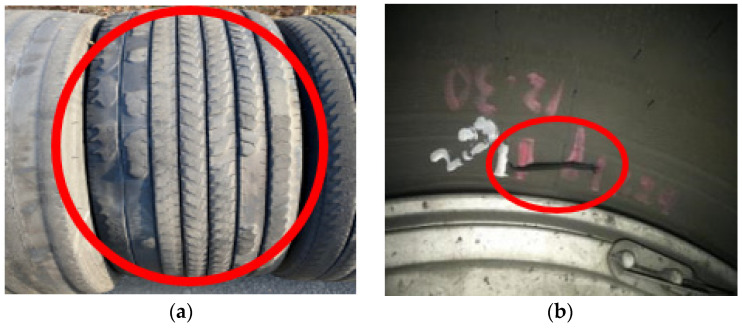
(**a**) Uniform wear, (**b**) Tire bead crack.

In Figure 1, it illustrates the abnormal wear and damage to the tires occurring during the operation of the light rail, while Table 1 presents the tire replacement status for Busan Metro Line 4.

Furthermore, relying on the subjective criteria of maintenance personnel can lead to excessive tire usage beyond the specified wear limits, increasing the risk of secondary damage to the vehicles during operation. This results in extended maintenance times, reduced operational availability, and poses a threat to punctual service schedules.

The current reliance on post-failure maintenance, where anomalies observed during inspections by maintenance personnel or abnormal physical occurrences during operation are addressed after they are detected, leads to a lack of technology for predicting and performing preventive maintenance to anticipate potential casualties to passengers resulting from wheel damage and vehicle destruction during operation. In other words, there is a deficiency in technology for proactive maintenance that could prevent casualties and damage [1,2,3,4]. Furthermore, there are issues related to passenger complaints due to wheel damage and derailments during operation, as well as economic losses incurred through increased maintenance costs and reduced operational efficiency [5,6,7].

Research is needed for technologies that can prevent and prevent accidents by diagnosing the condition of tires in advance through more precise and scientific preliminary condition classification and diagnosis technology for the wheels of railway vehicles. This study is a follow-up study to “A Study on Wheel Member Condition Recognition Using Machine Learning (Support Vector Machine) [8]”. In this study, the authors actually installed devices such as temperature/pressure/acceleration sensors, collection devices, and analysis program linkages to classify the condition of rubber tires on Busan Line 4 light-rail vehicles and used actual measurement data on operation. For the first time, actual measurements during operation were used. The use of data provides innovative differentiation of this study and high confidence in the results.

In this study, key factors were selected through principal factor analysis of collected light-rail operational data, and data preprocessing was performed to generate the final training dataset [8]. Using a deep learning-based 1D–CNN technique, a ternary classification method (Good, Fair, Poor) for light-rail vehicle tire conditions is proposed. The results showed a high accuracy of 99.4% in condition classification. This research is part of a national research and development project supported by the South Korean Ministry of Land, Infrastructure, and Transport. Based on the research findings, there are plans to implement practical applications of tire condition prediction equipment for light rail, including the Busan Metro Line 4, currently in operation, and newly established lines throughout the country.

## 2. Technological Trends in Predicting Abnormalities in Railway Vehicle Wheels

Tire wear is a critical factor that influences tire lifespan and occurs due to the detachment of rubber material when frictional forces between the road surface and the tire surface exceed the strength of the rubber material [9]. In this chapter, the authors introduce relevant studies related to the content presented in this paper.

One of the recent studies aimed at verifying tire wear performance involves the use of intelligent tires. These tires are equipped with temperature, pressure, and acceleration sensors inside the tire to estimate tire wear [10,11].

‘Supplementary Magnetic Tests for Railway Wheel Sets’, a framework based on magnetomechanical effects and magnetic processes resulting from the magnetic characteristics of wheel-set aging was proposed [12].

‘Acoustic Emission Monitoring of Wheel Sets on Moving Trains’, they established an acoustic emission monitoring system for train wheels by mounting acoustic emission sensors on the rails [13].

‘Wheel Defect Detection with Machine Learning’ conducted research on automatic detection of wheel vertical force-based defects through sensor data using two machine-learning techniques [14].

‘Train Wheel Condition Monitoring via Cepstral Analysis of Axle Box Accelerations’ provides an approach to wheel condition monitoring based on Cepstral analysis of Axle-Box Accelerations (ABA). It allows monitoring of wheel circumference and the consequent wheel wear, with the peak amplitude in the cepstrum domain indicating the severity of potential wheel defects [15].

‘Development and testing of an automatic remote condition monitoring system for train wheels’ proposes an automatic optical system designed to detect wheel defects in operational railway vehicles. It describes the process of capturing high-resolution and high-quality images of wheel treads and flanges, suitable for defect detection [16].

In ‘Condition monitoring of train wheel wear and track forces: a case study’ a study was conducted to investigate the correlation between wheel and rail wear rates and dynamic forces in order to determine cost-effective wheel maintenance intervals [17].

In ‘Infrared Diagnostics of Cracks in Railway Carriage Wheels’, a method for detecting cracks in railway carriage wheels is proposed based on the surface temperature distribution of the wheel during wheel disc heating, using thermal imaging and infrared cameras [18].

‘Real-Time Train Wheel Condition Monitoring by Fiber Bragg Grating Sensors’ proposes a real-time system for monitoring wheel defects using Fiber Bragg Grating sensors. It measures and processes track deformation responses during wheel–rail interactions to create a condition index that directly reflects the wheel’s state [19].

‘Automatic clustering-based approach for train wheels condition monitoring’ presents an unsupervised methodology for identifying railway wheel flats. It is based on the evaluation of acceleration measured on the rails during train passage and discusses the application of a two-step procedure [20].

Research on classifying the wheel condition of railway vehicles is actively conducted, but most studies focus on steel wheels, and research on rubber wheels is inadequate. Furthermore, experiments for classifying the condition of rubber wheels mostly involve automobiles. However, this paper, as a follow-up study to ‘A Study on Wheel Member Condition Recognition Using Machine Learning (Support Vector Machine)’, installed sensors on rubber wheels of in-service light-rail vehicles to collect data such as temperature, pressure, and acceleration. Through factor analysis and preprocessing, training data were generated. Based on the created training data, this paper proposes a method using deep learning to classify the real-time condition of light-rail rubber wheels [8].

## 3. Installation of Measuring Equipment

To enable predictive classification of tire conditions in Busan Metro Line 4, wireless sensors (temperature, pressure, internal acceleration) were installed inside the railway vehicle’s wheel sets. Additionally, measurement equipment was installed on the wheel truck, including axle acceleration, speed, behavior, wireless receivers, and data collection devices.

**Figure 2:**
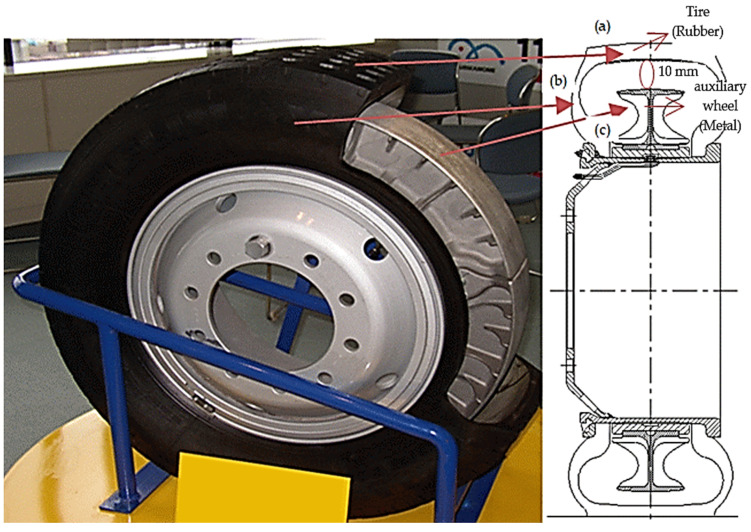
Tire structure of railway vehicle wheels.

Figure 2 provides an illustration of the structure of a light-rail vehicle wheel for better understanding. Figure 2, an auxiliary wheel made of metal material is present on the tread surface (a) and the side surface (b) of the tire to withstand tire damage. However, the internal space between the tire reinforcement (c) and the tire itself is very narrow, approximately 10 mm, making it physically tight for the installation of temperature/pressure/acceleration sensors and wireless transmitters inside the tire. Moreover, during the operation of the light-rail vehicle, the tire not only rotates at a very high speed but also undergoes deformation of the side surface (b) as it rolls, posing significant challenges and difficulties in installing measurement equipment. Additionally, the temperature/pressure sensors and the wireless transmitter for temperature/pressure are installed as an integral unit, with a portion cut into the top surface of the tire reinforcement. The internal acceleration sensor inside the tire is attached to the inner surface of the rubber material on the tread surface (a) to ensure the reliability of the internal acceleration data. Wireless transmission of data into the vehicle cabin was necessary. However, the construction of railcar vehicles, with their undercarriage designed as a shielding structure, posed difficulties in achieving wireless reception under the railcar. Moreover, the dynamic nature of tire operation required the collection of operational data, making data acquisition a demanding task. Despite these challenges, this research successfully collected real-time operational data for light-rail vehicles for the first time. This innovative approach offers a high level of differentiation, reliability, and practicality based on the collected data.

**Figure 3:**
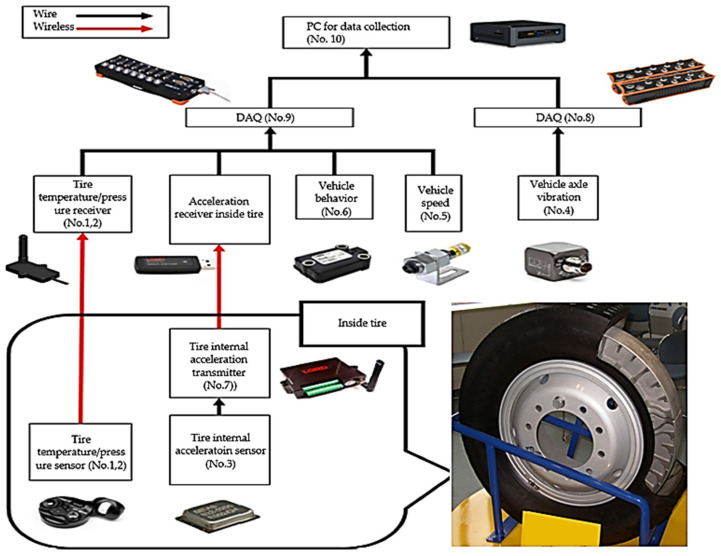
Measuring equipment installation signal diagram for railway vehicle wheels.

**Figure 4:**
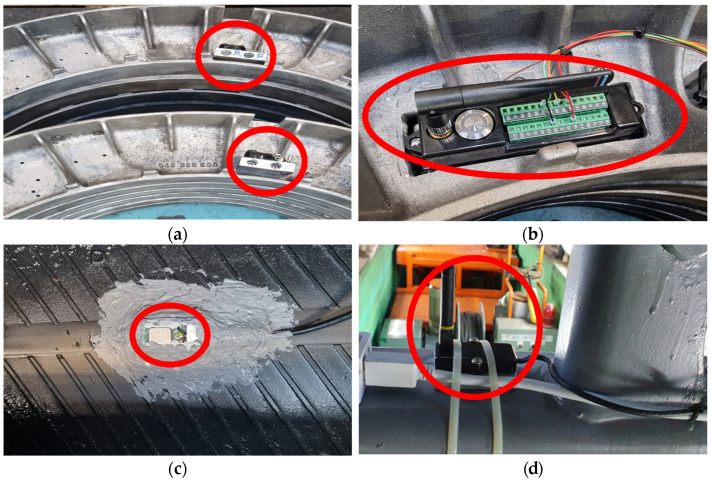

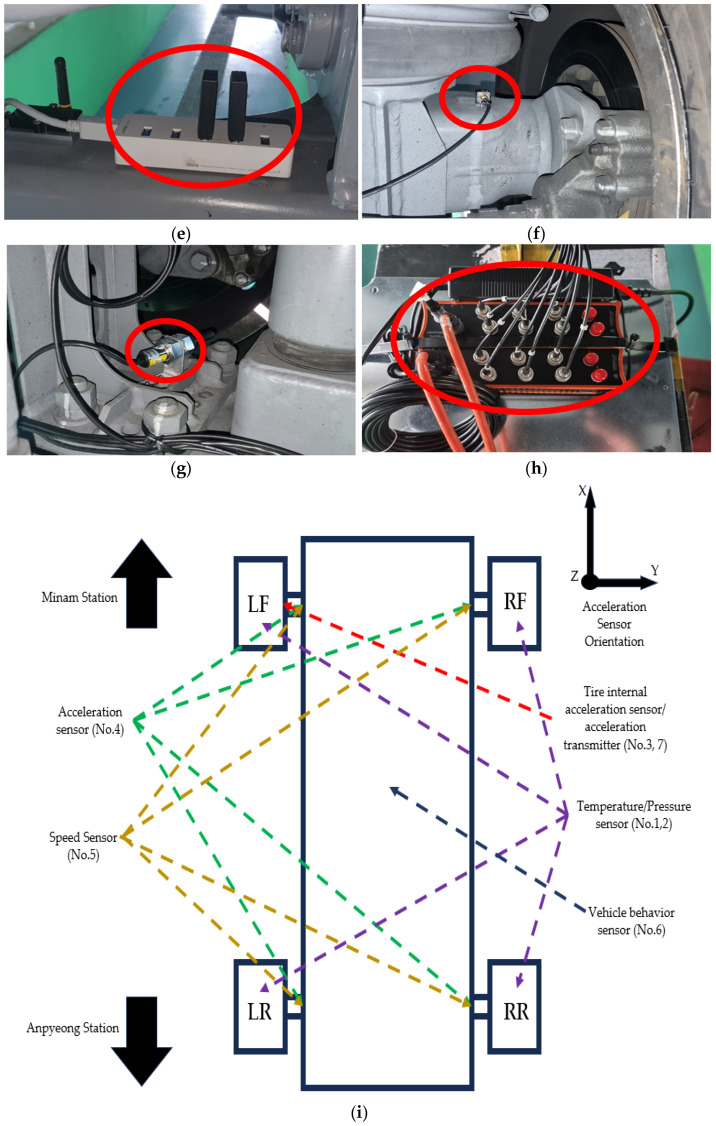
Installation of measuring equipment inside railway vehicles: (**a**) Temperature/pressure sensor installation (No.1,2), (**b**) Installation of acceleration sensor transmitter inside tire (No.7), (**c**) Installing acceleration sensor inside tire (No.3), (**d**) Installation of tire internal temperature/pressure sensor receiver (No.1,2), (**e**) Installing the acceleration sensor receiver inside the tire, (**f**) Axial acceleration sensor installation (No.4), (**g**) Speed sensor installation (No.5), (**h**) Data acquisition device installation (No.8), (**i**) Location of sensors installed on railway vehicles

Table 2 lists information about the sensors and measurement equipment installed on the railway vehicle, and Figure 3 illustrates the signal flow between the sensors and measurement equipment installed on the railway vehicle for easy comprehension. Additionally, Figure 4 provides a clear representation of the sensor locations. 

## 4. Analysis of Key Factors for Deep Learning-Based Tire Condition Classification Research

### 4.1. Correlation Analysis between Tire Temperature/Pressure/Acceleration of Railway Vehicle Wheels

**Figure 5:**
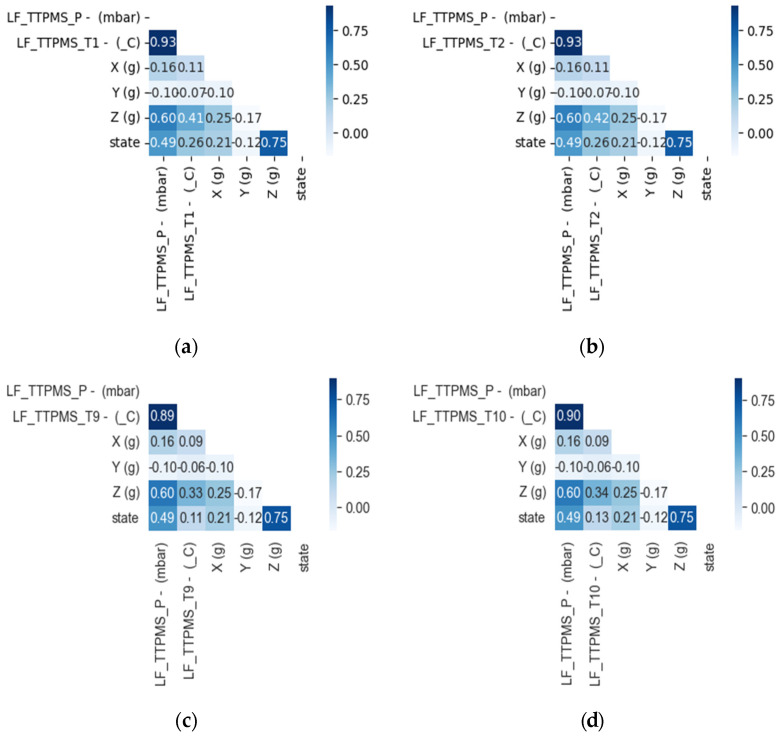

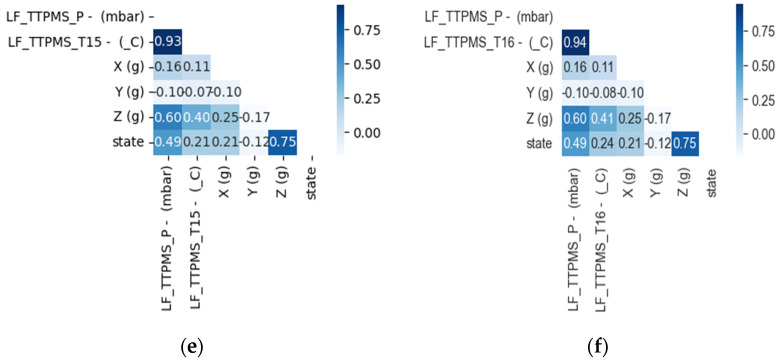
Tire temperature/pressure/acceleration correlation analysis: (**a**) T1 point correlation coefficient analysis heatmap, (**b**) T2 point correlation coefficient analysis heatmap, (**c**) T9 point correlation coefficient analysis Heatmap, (**d**) T10 point correlation coefficient analysis heatmap, (**e**) T15 point correlation coefficient analysis heatmap, (**f**) T16 point correlation coefficient analysis heatmap.

**Figure 6:**
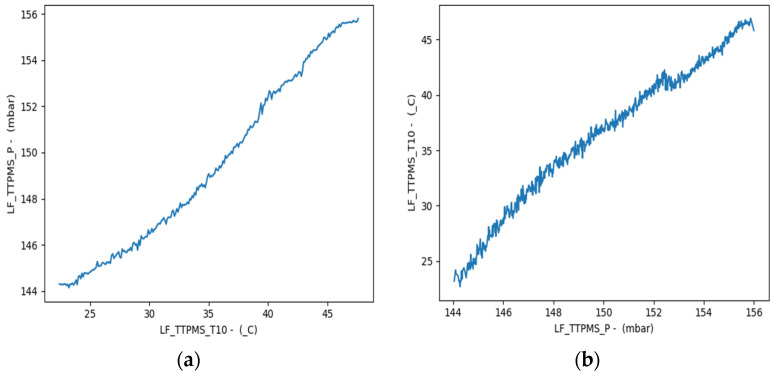
(**a**) Linearity relationship based on tire temperature, (**b**) Linearity relationship based on tire pressure.

To conduct the classification study of the tire conditions in the Busan Metro Line 4 railway vehicle, the correlation between temperature, pressure, and acceleration, based on actual measurement data, was analyzed. The analysis of the correlation for each of the 16 internal temperature points in the tire yielded results similar to Figure 5. The magnitude of the absolute value of the correlation coefficient indicates the degree of linearity, with the sign indicating the direction of the linear relationship. As the absolute value of the correlation coefficient approaches 1 or −1, the relationship between the two variables is stronger, while values closer to 0 indicate a weaker relationship. The correlation analysis showed that the internal temperature and air pressure within the railway vehicle wheel’s tire are directly proportional and demonstrated the highest correlation. Figure 6 is a graph showing the linear relationship between tire temperature and pressure. Figure 6a shows the linear relationship based on temperature, and Figure 6b shows the linear relationship based on pressure.

### 4.2. Tire Condition Classification Analysis of Tire Temperature, Pressure, and Acceleration Factors of Railway Vehicle Wheels

A classification analysis of temperature/pressure/acceleration factors and tire conditions, which are actual tire operation data of Busan Line 4 railway vehicle wheels, was performed. Status 0/1/2 refers to the status according to the tire tread depth, which is the dependent variable of this study. The standard was set to tire condition good(0): tread 15 mm, tire condition fair(1): tread 8 mm, tire condition poor(2): tread 1.6 mm.

**Figure 7:**
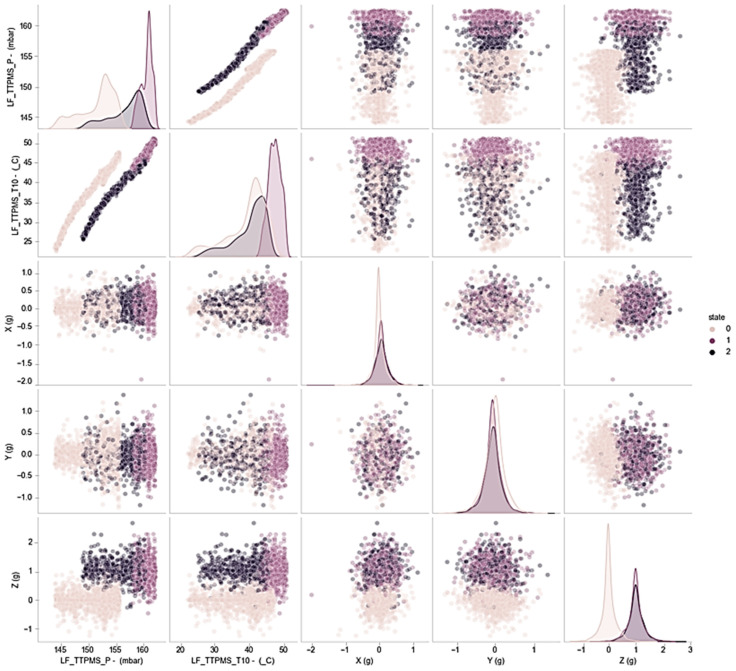
Scatter plot of classification analysis of tire temperature/pressure/acceleration and tire condition at point T10.

Figure 7 depicts the results of the classification analysis conducted at the T10 location, which served as the final model training data. The analysis reveals that for the tire conditions fair(1) and poor(2), there is a significant overlap in the data distribution.

The scatterplot analysis of tire condition classification based on X-axis acceleration and Y-axis acceleration shows that, overall, the classification is not well defined. However, significant classification can be observed between Z-axis acceleration and pressure, as well as between Z-axis acceleration and temperature.

## 5. Design of a 1D–CNN Model for Deep Learning-Based Tire Condition Classification Research on Railway Vehicle Wheels

### 5.1. Design of 1D–CNN Model for Research on Tire Condition Classification of Railway Vehicle Wheels

The 1D–CNN model is well-suited for sensor/signal data analysis and has shown better performance compared to other deep-learning models. It excels in identifying relatively simple patterns within the data and is also suitable for time series data analysis. Therefore, in this study, the authors designed a 1D–CNN model.

**Figure 8:**
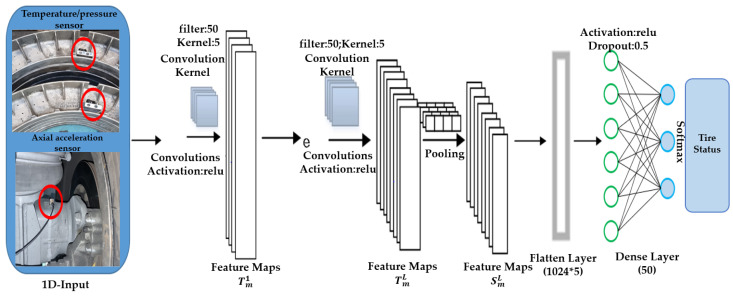
1D–CNN-based tire condition classification using light-rail driving measurement data.

In this study, a model with five independent variables, which are the internal tire temperature, pressure, and 3-axis accelerations (x, y, z), was designed for input factors related to railway vehicle wheel tires. Figure 8 is an illustration to aid in understanding the 1D–CNN-based tire condition classification using railway vehicle operational measurement data.

Convolution Layer 1: [filters] 50, [Kernel_size] 5, [Kernel_initializer] he_uniform,
[Input_shape] (5, 1), [Activation] relu.
Convolution Layer 2: [filters] 50, [Kernel_size] 1, [Kernel_initializer] he_uniform, [Activation] relu.
Convolution Layer 3: [filters] 50, [Kernel_size] 1, [Kernel_initializer] he_uniform, [Activation] relu.
Pooling Layer: MaxPooling1D [pool_size] 1, [padding] valid.
Dense Layer 1: [node_size] 100, [Activation] relu.
Dense Layer 1: [node_size] 100, [Activation] relu.
Dense Layer 1: [node_size] 100, [Activation] relu.
Output Layer: Dense [node_size] 3, [Activation] softmax.

The above description explains the architecture of the 1D–CNN-based tire condition classification model. Table 3 presents examples of independent variables for the input data of the 1D–CNN-based tire condition classification model.

### 5.2. Verification of 1D–CNN Model Hyperparameters for Learning Tire Condition Classification Model for Railway Vehicle Wheels

For the training of the tire condition classification model for railway vehicle wheels, hyperparameter validation tests were performed for the 1D–CNN model. The training involved adjusting the number of Convolution Layers and Dense Layers, as well as the number of nodes in the designed 1D–CNN model to validate the loss value and accuracy. Furthermore, in order to select the training data for the internal tire temperature measurement points, performance tests were conducted at locations T1 to T16. Hyperparameter validation tests revealed that the best performance was achieved with a Convolution Layer depth of 3, Dense Layer depth of 3, and 100 nodes in the Dense Layer, resulting in an accuracy of 98.65%. In the selection test for the tire’s internal temperature measurement point data, T10 exhibited the highest performance with an accuracy of 98.91%. Table 4 presents the results of the hyperparameter validation test for the 1D–CNN-based tire condition classification model, while Table 5 illustrates the results of the test for selecting the training data for the tire’s internal temperature measurement points.

### 5.3. 1D–CNN Final Learning Model for Research on Tire Condition Classification of Railway Vehicles

**Figure 9:**
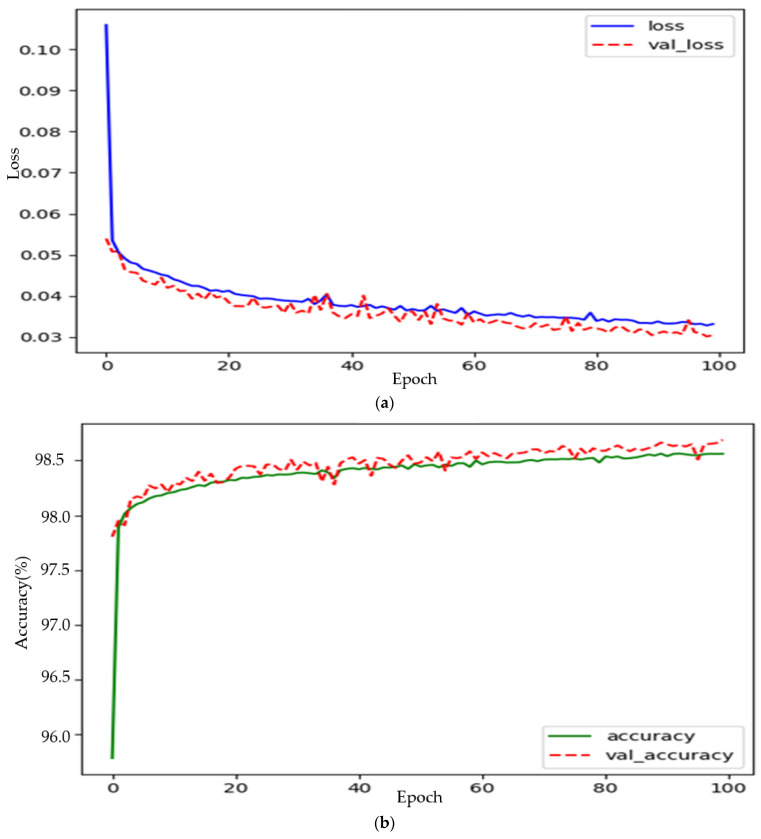
Tire condition classification model learning: (**a**) Loss graph, (**b**) Accuracy graph.

The training data were derived from actual measurements obtained by running two round trips on the Busan Line 4 between Anpyeong and Minam. These raw data included temperature, pressure, and acceleration information. The data were then processed to categorize the tire’s condition into “Good”, “Fair”, or “Poor” based on the tread depth, with the respective condition serving as the target value. Furthermore, the sensor data were collected at varying sampling frequencies, with acceleration data at 1000 Hz and temperature/pressure data at 100 Hz. To ensure synchronization, all data were downsampled to a common minimum frequency of 100 Hz. When comparing the downsized acceleration data at 100 Hz to the original 1000 Hz data, there was no discernible difference in the loss of information. Outlier data was additionally removed, the final learning data was selected, and the final model learning result showed the highest performance with accuracy of 98.91% at Epoch 100/Batch size 2048. Table 6 provides an explanation of the composition of the training dataset for the 1D–CNN-based classification of the tire condition of railway vehicle wheels. Table 7 displays the number of samples in the training dataset for the state classification model. Table 8 shows the training results. Figure 9 visualizes the loss and accuracy graphs for the tire condition classification training. Table 9 visualizes the performance of the algorithm.

During the research for this paper, actual tire data was collected for the light-rail vehicles on Busan Metro Line 4 operating between Anpyeong Station and Minam Station, covering two round trips. The tires were equipped in ‘Good’, ‘Fair’, and ‘Poor’ conditions, and data for each tire condition were gathered. After performing data preprocessing, including the removal of data outliers, the final dataset was composed of 1,567,706 training data points and 313,375 test data points. The 1D-CNN-based state prediction results, as shown in Table 9, were validated based on the predicted tire condition and real tire condition. In other words, as demonstrated in Table 9, the actual ‘Good’ tire condition was predicted with 100% accuracy. The ‘Fair’ condition tires were predicted with a probability of 98.75%, and the ‘Poor’ condition tires were predicted with a probability of 97.73%.

### 5.4. Improved Performance of Deep Learning-Based Tire Condition Classification Model

To enhance the performance of the 1D–CNN classification model used for Busan Line 4 tire-state classification, a 1D–CNN tuning model was designed. For this purpose, parameter optimization was conducted. To optimize hyperparameter values, parameter tuning was conducted using Bayesian Optimization, which exhibited faster speed and better cost-effectiveness compared to Grid Search and Random Search. Similar to the 1D–CNN-based classification model, a ternary classification was performed for tire conditions, where “Good” (0) corresponded to a tread depth of 15 mm, “Fair” (1) to 8 mm, and “Poor” (2) to 1.6 mm of tread depth. Approximately 900,000 training data points, 300,000 test data points, and 700,000 validation data points were used. As a result, better performance metrics than the 1D–CNN-Based model, including accuracy, recall, precision, and F1 Score, were achieved, and the details are summarized in Table 10 and Table 11.

### 5.5. Deep Learning-Based Tire Condition Classification Performance Evaluation of Railway Vehicle Wheels

In order to compare the performance of the tire-state classification research results based on the SVM (Support Vector Machine) and Random Forest algorithms from the study “A Study on Wheel Member Condition Recognition Using Machine Learning (Support Vector Machine)” with the results of the tire-state classification research based on 1D–CNN used in this paper, performance comparison was conducted using evaluation metrics including accuracy, recall, precision, and F1 Score. The results were summarized and presented in Table 12. Upon comparing the results, it can be observed that the accuracy of the tire-state classification based on the 1D–CNN-Based model is 98.9%, which is superior to SVM (Linear Kernel) 98.7%, SVM (RBF Kernel) 96.5%, and Random Forest 89.7%. Furthermore, it can be noted that for the optimization model achieved through hyperparameter tuning for performance enhancement of the 1D–CNN-Based model, the 1D–CNN tuning model exhibited the best performance with an accuracy of 99.4%.

In the future, the deep learning-based 1D–CNN model will be used to commercialize the tire condition diagnosis system for railway vehicles on Busan Line 4, and the new Busan urban railway routes such as Nopo–Yangsan Line, Sasang–Hadan Line, Hadan–Noksan Line and it is also scheduled to be applied to light-rail vehicles on Gwangju Metro Line 2.

## 6. Conclusions

This study proposes a practical method for the classification of rubber tire conditions on light-rail vehicle wheels, aiming for predictive maintenance. It is practical research grounded in safety monitoring and maintenance optimization for rubber tires on light-rail vehicles, with the potential for cost savings, improved safety, and the prevention of operational delays. Instead of relying on reactive post maintenance, this research introduces a proactive maintenance approach for rubber tires on light-rail vehicles, leveraging their advantages of high acceleration, deceleration capabilities, and precise stopping abilities.

In this study, pressure, temperature, and internal acceleration sensors were installed inside the rubber tires of Busan Line 4 railway vehicles. Measurement equipment was installed on the undercarriage of the vehicles to collect operational data, including vehicle axle acceleration, vehicle speed, and vehicle behavior, during real railway vehicle operations. The data collection process faced numerous physical and technical challenges, but the data was successfully collected.

An analysis was conducted on the collected data to derive key factors, resulting in the extraction of temperature, pressure, and acceleration data as factors suitable for tire condition classification. Among these factors, the Z-axis acceleration was identified as the factor with the highest impact on tire condition.

For each tire condition, data for training were generated using two round trips on the Busan Line 2 route. Data preprocessing steps, including sample frequency synchronization and outlier data removal, were carried out.

As a follow-up study to “A Study on Wheel Member Condition Recognition Using Machine Learning (Support Vector Machine)”, this research improves the performance of condition classification using deep learning instead of machine learning. A 1D–CNN model, suitable for processing sequence data, was primarily employed. SVM with Linear Kernel and RBF Kernel achieved accuracies of 98.7% and 96.5%, respectively, while Random Forest showed an accuracy of 89.7%. In contrast, the 1D–CNN used in this study achieved a high accuracy of 98.9%. Furthermore, a 1D–CNN tuning model, designed through Bayesian Optimization for hyperparameter optimization, demonstrated the highest accuracy of 99.4%, showcasing superior performance among the models.

This research enhances the learning and prediction performance of deep-learning algorithms using temperature, pressure, and 3-axis acceleration data as key factors. It provides a method for proactive preventive maintenance of rubber tires on railway vehicle wheels, moving away from relying on the occurrence of physical anomalies or post maintenance checks.

This practical technological enhancement contributes to cost savings in light-rail vehicle operations, enhances safety, and helps prevent operational delays. By providing foundational technology for practical preventive maintenance methods, this research is poised to contribute to the widespread adoption of light rail, considering its economic advantages, in future urban railway construction projects.

## Figures and Tables

**Table 1:** Busan Line 4 tire replacement status.

Year		2013	2014	2015	2016	2017	2018	2019	Sum
Abnormal Wear Replacement	Crack, Uneven wear [ea]	66	90	93	98	67	42	40	496 (48.0%)
	Tire Damage [ea]	1	1	0	1	9	14	12	38 (3.7%)
	Subtotal [ea]	67	91	93	99	76	56	52	534 (51.6%)
Maintenance Replacement [ea]		47	79	42	55	129	70	78	500 (48.4%)
Sum [ea]		114	170	135	154	205	126	130	1034

**Table 2:** Measuring equipment.

NO	Category	Driving Condition	Location (Quantity)	Measurement Purpose	Sensor Model	Manufacturing Company	Nation
**1**	Tire pressure	Driving (by season)	Inside tire (4set)	Air pressure and temperature changes depending on external environment and tire condition	TTPMS–V2	IZZ RACING	USA
**2**	Tire temperature						
**3**	Internal acceleration	Driving		Acceleration and vehicle speed changes depending on air pressure, temperature, load, and wear	3038 –200	TE	
**4**	Vehicle axle acceleration		Railcar axle (4set)		BST 903	DUETTO	GERMANY
**5**	Vehicle speed		Tire wheel (4set)		ROLS–W	MONARCH	USA
**6**	Vehicle behavior		Floor outside the room (4set)	Vehicle behavior measurement	ML5 –AR	Lord	
**7**	Wireless transmitter		Inside tire (4set)	Tire internal acceleration data	V –link 200	Lord	
**8**	DAQ (acceleration)		Floor outside the room (2set)	8 channel 2set (X, Y, Z) × 4	KRYPTON	DEWE Soft	AUS TRIA
**9**	DAQ (pressure)		Floor outside the room (1set)	Temperature/pressure: CAN I/F Internal acceleration: Wireless IMU: CAN I/F Tire speed: 4channel	DEWE–43		
**10**	PLUG–IN		Collectible pc (1copy)	Tire internal acceleration (wireless) measurement program	DEWE Soft –op –Lord		

**Table 3:** Example of 1D–CNN-based tire condition classification model input data and independent variables.

LF_TTPMS_P–[mbar]	LF_TTPMS_P–[_C]	X[g]	Y[g]	Z[g]
9950.02	22.700001	−0.016261	0.023190	−0.039249
9950.02	22.700001	−0.017589	0.023169	−0.038865
9950.02	22.700001	−0.016466	0.021973	−0.040368
9950.02	22.700001	−0.017130	0.022423	−0.038767
9950.02	22.700001	−0.017928	0.022153	−0.040424
9950.02	22.700001	−0.016996	0.022042	−0.039718
9950.02	22.700001	−0.017674	0.022568	−0.038404
9950.02	22.700001	−0.015851	0.020735	−0.040081
9950.02	22.700001	−0.017201	0.021579	−0.038459
9950.02	22.700001	−0.016798	0.020874	−0.039165

**Table 4:** Hyperparameter verification test of 1D–CNN-based tire condition classification model.

Epoch	Convolution Layer Depth	Dense Layer Depth	Dense Layer Node	Loss	Accuracy [%]
50	1	3	10	0.05	97.93
		3	100	0.05	97.93
		1	10	0.05	97.88
		1	100	0.05	98.00
		1	1024	0.05	97.99
	2	3	10	0.05	97.97
		3	100	0.03	98.27
		1	10	0.05	97.99
		1	100	0.04	98.01
		1	1024	0.04	98.04
	3	3	10	0.05	98.02
		3	100	0.03	98.65
		1	10	0.05	97.94
		1	100	0.04	98.04
		1	1024	0.04	98.16

**Table 5:** Learning data selection test at tire internal temperature measurement point.

Tire Location	Temperature Measurement Point	Early Stopped	Loss	Accuracy [%]
LF	T1	100	0.13	92.71
	T2	100	0.13	92.96
	T3	100	0.12	93.10
	T4	100	0.13	93.09
	T5	100	0.12	93.01
	T6	100	0.12	93.24
	T7	100	0.10	94.37
	T8	100	0.06	96.81
	T9	100	0.03	98.36
	T10	100	0.02	98.91
	T11	100	0.03	98.72
	T12	100	0.03	98.5
	T13	100	0.03	98.57
	T14	100	0.07	96.35
	T15	100	0.11	94.00
	T16	100	0.13	93.07

**Table 6:** Tire condition classification learning data set configuration.

Category	Unit	Detail
Input variable	Pressure [pa]	Pressure value
	Temperature [C]	10th out of 16 branches
	Acceleration [g]	X axis: direction
		Y axis: transverse
		Z axis: longitudinal
Output variable	–	Tire condition ternary classification Good (tread depth: 15 mm) Fair (tread depth: 8 mm) Poor (tread depth: 1.6 mm)

**Table 7:** 1D–CNN-based tire condition classification model training data for railway vehicles.

Tire Condition	Anpyeong to Minam (1)	Minam to Anpyeong (2)	Anpyeong to Minam (3)	Minam to Anpyeong (4)	Total Data
Good	156,604	156,604	152,935	154,212	620,355
Fair	123,236	123,790	123,234	123,865	494,250
Poor	114,829	114,884	114,148	114,671	458,532

**Table 8:** Final learning result of 1D–CNN-based tire condition classification model for railway vehicle wheels.

Model	Epoch	Early Stopped	Batch Size	Loss	Accuracy [%]
1D–CNN	100	100	2048	0.03	98.91
		86	1024	0.03	98.43
		87	512	0.03	98.54
	300	87	2048	0.03	98.62
		53	1024	0.3	98.56
		78	512	0.3	98.59
	500	81	2048	0.3	98.61
		81	1024	0.3	98.51
		68	512	0.3	98.49

**Table 9:** 1D–CNN-based tire condition prediction result for railway vehicle wheel Confusion Matrix.

		Predicted Tire Condition		
		Good	Fair	Poor
Real tire condition	Good	123,563	0	0
	Fair	0	97,314	1228
	Poor	0	2075	89,195

**Table 10:** Hyperparameter optimization values.

Hyperparameter	Optimization Value
Learning Rate	0.17
Layer Depth	2
Batch Size	577
Epoch	821
Filter	78
Activation Function	ReLu
Kernel Initialize	Random–uniform
Optimizer	SGD

**Table 11:** Comparison results of tire condition classification performance of railway vehicle wheels based on 1D–CNN.

Model	Accuracy	Recall	Precision	F1 Score
1D–CNN Based	98.9%	98.8%	98.8%	98.8%
1D–CNN Tuning	99.4%	99.4%	99.4%	99.5%

**Table 12:** Performance evaluation comparison results.

Model	Performance Evaluation Indicators			
	Accuracy	Recall	Precision	F1 Score
SVM (Linear Kernel)	98.7%	98.7%	98.8%	98.7%
SVM (RBF Kernel)	96.5%	96.5%	96.9%	96.7%
Random Forest	89.7%	89.7%	92.1	90.9%
1D–CNN-Based	98.9%	98.8%	98.8%	98.8%
1D–CNN Tuning	99.4%	99.4%	99.4%	99.5%

## Data Availability

Data are contained within the article.

## Abbreviations

These are the abbreviations and nomenclature used in this paper.

CNN	Convolution Neural Network
SVM	Support Vector Machine
RBF Kernel	Radial Basis Function Kernel
Auxiliary wheel	Auxiliary wheels present in the tires of light-rail wheels
Railcar axle	Central axis around which the train’s wheels rotate
Bayesian Optimization	One of the deep-learning parameter optimization methods
Light rail	Lighter railway vehicle than existing railway vehicles
Wheel truck	Wheel mounting location
Tire bead	Part where the tire and wheel (rim) come into contact with each other
Tread surface	Part that is in direct contact between the wheels and the road
